# An optimization approach for freeway network coordinated traffic control and route guidance

**DOI:** 10.1371/journal.pone.0204255

**Published:** 2018-09-28

**Authors:** Minghui Ma, Shidong Liang

**Affiliations:** 1 Automobile Engineering College, Shanghai University of Engineering Science, Shanghai, China; 2 Business School, University of Shanghai for Science and Technology, Shanghai, China; National Taiwan University of Science and Technology, TAIWAN

## Abstract

Traffic congestion in freeway network is a prevalent transportation problem and leads to a strong degradation of freeway network facilities. This paper proposes a new coordinated optimization approach for freeway network, aiming to relieve the traffic congestion in freeway network equipped with the coordinated traffic controllers. The challenge is how to solve the problem between the traffic control goals of each path and those of the network. In this paper, the bi-level programming theory is used to address this problem. More exactly, in the path level, the optimal control model is designed to relieve congestion in each path of the test network. Furthermore, in the network level, the optimal model considers the traffic equilibrium and cost optimization. The efficiency of the proposed approach is tested on a well-known traffic network calibrated by the field test data of freeway network. The results show that the proposed approach has positive effects in balancing traffic load and improving the service level of freeway networks.

## Introduction

Traffic congestion in freeway network becomes more noticeable in modern societies, introducing negative effects for a sustainable development of the Intelligent Transport System (ITS), such as the declining public transport service level and the increasing traffic costs [1]. Variable speed limits (VSL) is a new mainline traffic control method and is widely applied on freeway mainlines to solve the traffic congestion problem, such as in Hadiuzzaman and Qiu [2]. The VSL values are the core of the VSL control, calculated by diverse traffic conditions involving traffic volume and traffic density. Instead of using static speed limits, many studies on the VSL control present a common result that the VSL control can enhance traffic safety, decrease drivers’ complain, and improve the obey rate of drivers. This has been investigated by Moghadam [3], Hegyi et al. [4] and Li et al. [5]. According to these studies, several views exist on the use of VSL control. Heydecker and Addison [6] emphasized the homogenization effect. In contrast, Abdel-Aty and Wang [7], Yang et al. [8], and Yang and Hegyi [9] mainly focused on the prevention of freeway traffic flow breakdown and maximization of traffic flow. To apply the VSL control strategies in the mainline area without considering the influence of traffic outflows from on-ramp, this approach cannot effectively solve the traffic congestion problem in merging region during peak periods[10, 11]. The congestion may transfer to the upstream mainline and the related freeway network, if the VSL control or/and ramp metering in the problem path is applied alone during peak periods.

To overcome these problems mentioned above, a coordinated approach, involving ramp metering, VSL control, and route guidance, has been gradually paid more attention (see e.g. Wang and Papageorgiou[12], Liu et al.[13], and Pasqual et al. [14]).To mitigate the interference of the on-ramp flows and relieve congestion in freeway network, Pasquale et al.[15] and Kotsialo et al. [16, 17] attempted to fit the freeway network control problem in the format of a discrete-time optimal control problem and solve it by using both integrating ramp metering and route guidance. Karimi et al. [18] proposed an integrated control approach, involving VSL control, ramp metering and route guidance. However, these studies only tested the efficiency of coordinating ramp metering and route guidance. Moreover, most traffic control methods pay little attention to the detailed analysis of the relationship between the traffic-controlled objective and route guidance objective, which are therefore not suitable in certain conditions (such as there is a specific problem path existed in the freeway network).

In this paper, we propose an optimization approach based on a bi-level programming optimal model to relieve the congestion in the freeway network, integrating VSL control, ramp metering, and route guidance. The contributions of this paper can be summarized as follows.

- A coordinated optimization approach is proposed to relieve the traffic congestion in a freeway network on the basis of the bi-level programming model.
- The upper-level programming model mainly handles the traffic loading distribution equilibrium problem and the total travel time in the network. Since the traditional definition of traffic load ignores some special conditions (e.g. bottleneck region and merging region) existing in the path, a novel traffic load definition is given as the ratio between the maximum traffic flow among each section and the minimum capacity of each section in the same path.
- The lower-level programming model coordinates the VSL control and ramp metering. We consider a variety of situations, involving two kinds of common problem paths and comprehensive conditions. Furthermore, we select both travel time and traffic flow in each path as the control goal to establish the objective function.

The rest of this paper is organized as follows. Section 2 contains macroscopic traffic flow model and the extension model. The bi-level optimal model is formulated in detail in Section 3, while Section 4 contains some illustrative and experimental evaluations obtained by different control strategies. Finally, in Section 5, conclusions and some topics for further research are summarized.

## Macroscopic traffic flow model

### Traffic flow model on normal section

A foundation macroscopic traffic flow model described in detail by Messmer and Papageorgiou [19] and Kotsialos et al. [20] is applied in this paper to descript the traffic flow parameters. The macroscopic traffic flow model is used for the description of freeway traffic flow.

**Fig 1:**
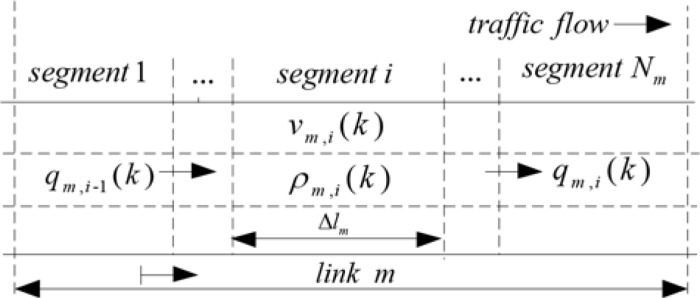
Discretized freeway link.

To introduce the operation principle of METANET model, we select one link as an example section. The discrete time step is denoted by *T* (typically, *T* = 10*s*). Take the link *m* as an example, which is divided into *N*_*m*_ segments with the length of Δ*l*_*m*_, where Δ*l*_*m*_ is given by the product of free-flow velocity *v*_*f*,*m*_, see Fig 1. The discrete-time instant can be expressed by *t* = *kT*. The macroscopic traffic flow parameters are defined as follows. The traffic density *ρ*_*m*,*i*_(*k*) represents the number of vehicles in single line of segment *i* of link *m* during period *k*. The traffic speed *v*_*m*,*i*_(*k*) represents the average speed of the vehicles in segment *i* of link *m* during period *k*. The traffic volume *q*_*m*,*i*-1_(*k*) represents the number of vehicles inflowing segment *i* of link *m*. *q*_*m*,*i*_(*k*) represents the number of vehicles outflowing of segment *i* of link *m*.

The macroscopic traffic flow model expresses the traffic parameters on freeway networks at a certain space-time domain. For each segment *i* of link *m* at each time step *k*, the following equations are applied.

$$q_{m,i}(k)=\rho_{m,i}(k)v_{m,i}(k)\lambda_{m},$$ (1)

$$\rho_{m,i}(k+1)=\rho_{m,i}(k)+\frac{T}{\Delta l_{m}\lambda_{m}}\left[q_{m,i-1}(k)-q_{m,i}(k)\right],$$ (2)

$$v_{m,i}(k+1)=v_{m,i}(k)+\frac{T}{\tau }\left\{V\left[\rho_{m,i}(k)\right]-v_{m,i}(k)\right\}+\frac{T}{\Delta l_{m}}\left[v_{m,i-1}(k)-v_{m,i}(k)\right]v_{m,i}(k)-\frac{\upsilon T}{\tau \Delta l_{m}}\frac{\rho_{m,i+1}(k)-\rho_{m,i}(k)}{\rho_{m,i}(k)+\kappa },$$ (3)

$$V\left[\rho_{m,i}(k)\right]=v_{f,m}\exp\left[-\frac{1}{\alpha_{m}}{\left(\frac{\rho_{m,i}(k)}{\rho_{cr,m}}\right)}^{\alpha_{m}}\right],$$ (4)

where *ρ*_*cr*,*m*_ represents the critical density of the link *m* (the density at which the traffic flow reaches the capacity of link *m Q*_*cap*,*m*_) and *α*_*m*_ represents the model parameter of Eq (4) which expresses a nonlinear relationship between the traffic density and speed. In addition, *τ*, *υ* and *κ* represent constant parameters determined by the traffic system, driver’s behavior, geometry characteristic of link *m*, etc. Furthermore, the average speed of the segment *i* in link *m* is shown in Eq (3) which is limited by the minimum velocity *v*_min_. A slack movement, which is considered in Eq (3), includes the expected velocity, the velocity changes caused by the inflow *q*_*m*,*i*−1_(*k*), and speed increase (or decrease).

For seeking the reason that the mainstream speed decreases caused by ramp confluence, it is necessary to consider the traffic operation in merging region of the mainline and ramp. When there is an on-ramp on freeway, merging phenomena caused by on-ramp traffic flow and upstream mainline traffic flow appears in merging region and the range can be described by the term

$$-\frac{\delta Tq_{0}(k)v_{m,1}(k)}{L_{m}\lambda_{m}(\rho_{m,1}(k)+\kappa )},$$ (5)

where *δ* represents a constant parameter.

If there is a lane drop, the velocity change caused by weaving phenomena can be expressed as

$$-\frac{\phi T\rho_{m,N_{m}}(k)v^{2}{}_{m,N_{m}}(k)\left(\lambda_{m}-\lambda_{m+1}\right)}{L_{m}\lambda_{m}\rho_{crit,m}},$$ (6)

where *ϕ* represents the model parameter and *λ*_*m*_ − *λ*_*m*+1_ represents the number of dropped lanes.

### The extension model considering VSL

In order to describe the traffic flow evolution under VSL control, we should analyze the traffic characteristics under VSL control. The differences of the traffic operational mechanism whether the road traffic adopts VSL control are described as follows. If the VSL control is adopted, under the free-flow condition, the traffic speed is expressed by the limit speed *v*_*vsl*_ instead of the free-flow speed *v*_*f*_, where the limit speed value has to be less than the free-flow speed value, whereas the traffic follows self-organization operating under no special control condition. When the traffic condition is congestion or traffic density is higher than the critical density, the traffic under VSL control keeps a steady and regular operation on account of drivers have to act up to the VSL values to their vehicles. When the traffic density is close to the jam density *ρ*_*J*_, the vehicles have to obey self-organizing operation instead of the control strategy.

According to the discussions above, the desired speed variation caused by the VSL control can be expressed as a function of traffic density. Therefore, the desired speed under the VSL control can be given by

$$V_{m,i}\left[\rho_{m,i}(k)\right]=\min\left(v_{f,m}\exp\left[-\frac{1}{\alpha_{m}}{\left(\frac{\rho_{m,i}(k)}{\rho_{cr,m}}\right)}^{\alpha_{m}}\right],\eta V_{vsl,m}(k)+(1-\eta )v_{f,m}\exp\left[-\frac{1}{\alpha_{m}}{\left(\frac{\rho_{m,i}(k)}{\rho_{cr,m}}\right)}^{\alpha_{m}}\right]\right),$$ (7)

where *v*_*vsl*,*m*,*i*_(*k*) represents the speed limit value of segment *i* of link *m* during period *k* and *η* represents the parameter that drivers obey the VSL control [21, 22]. Especially, if *η* = 1, all the drivers obey the VSL values; else *η* = 0, all the drivers do not follow the VSL values; else, 0 < *η* < 1, a part of drivers obey the VSL values. In addition, either on-line or off-line traffic parameters can be obtained through the formulas mentioned in this section, which is possible to establish the following integrated control model as a fundamental for traffic flow description.

## Bi-level programming formulation of the freeway network

### Bi-level programming optimal model

The bi-level programming optimal model is widely employed for the optimal control and decision problem [23]. Bi-level programming optimal theory enables the decision manager to achieve the optimal control system by analyzing the theoretical relationship between the upper-level programming system and the lower-level programming system. The bi-level programming optimal model can be shown as follows.

The upper-level programming model

$$\begin{array}{l} \min J_{OU}[u,k]=\sum_{k=0}^{k_{p}-1}\Phi (x(k),u(k),v(k),d(k),k)\\ s.t.\\ x(k+1)=F[x(k),u(k),v(k),d(k),k]\\ H(x(k),u(k),v(k),d(k),k)\leq 0\\ k=1,2,\ldots ,k_{p} \end{array},$$ (8)

where *v*(*k*) = *v*(*u*,*k*) is determined by the lower-level programming model.

The lower-level programming model

$$\begin{array}{l} \underset{}{\min}J_{OL}[v,k]=\sum_{k^{\prime }=0}^{k_{p}-1}\varphi (x(k),u(k),v(k),d(k),k)\\ s.t.\\ x(k+1)=f[x(k),u(k),v(k),d(k),k]\\ g(x(k),u(k),v(k),d(k),k)\leq 0\\ k=1,2,\ldots ,k_{p} \end{array},$$ (9)

where *J*_*OU*_[*u*,*k*] and *J*_*OL*_[*v*,*k*] are the upper-level programming model and the lower-level programming model, respectively; *u* represents the decision vector of the upper-level decision makers, which is influenced by the objective vector of the lower-level decision vector *v*; Φ and *φ* are arbitrary functions, and *d* represents the disturbance variable; *H* and *g* represent the constraint sets of the upper-level decision vector and the lower-level decision vector, respectively.

**Fig 2:**
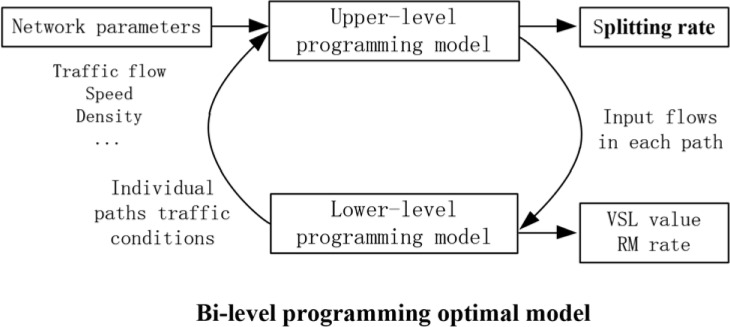
The proposed control system.

In this paper, the core concept of the optimal control approach includes two aspects. The first aspect is to alleviate the congestion in the problem path via VSL control and ramp metering. The other is to use route guidance to adjust the traffic flow distribution at the freeway network. The bi-level programming optimal model can analyze the decision problem of the subordinate construction and solve the coupling among different traffic control approaches that belong to different levels. Hierarchical partitioning between freeway traffic control and route guidance meets the demand and concept of a bi-level programming optimal approach. Specifically, in a coordination traffic system, the traffic load balancing and the total travel time minimum are selected as the optimal control objective functions of the upper-level programming system. The lower-level programming system establishes the optimal control model in paths, aiming to relieve the negative effects of the road traffic caused by traffic congestion. Furthermore, the coordinated control strategy proposed in this paper is shown as Fig 2.

### Formulation of the upper-level optimal model

The upper-level programming model involves two important factors, the expected benefits of drivers and the freeway network system. The travel time parameter attracts much attention among the expected benefits of driver, as it plays a significant role in the process of driver route selection. Hence, we select travel time as the evaluation index of traffic customer satisfaction and a sub-control objective of the upper-level optimal model. Furthermore, considering the traffic load in each path of freeway network, traffic managers generally balance the traffic distributions via traffic guidance. A change of the travel time can influence drivers’ route choice and traffic distribution in network because drivers pay more attention to the travel time. In this section, the first objective of the upper-level programming model mainly considers the travel time minimum, aiming to enhance the efficiency of the whole freeway network and improve the traffic load distribution equilibrium. The objective function of the upper-level programming of travel time can be presented as follows:

$$\min S_{TTT}=\sum_{k\in K}\sum_{i=1}^{p^{\prime }}F_{l_{i}}(x_{l_{i}}(k),u_{l_{i}}(k),v_{l_{i}}(k),d_{l_{i}}(k),k),$$ (10)

where *S*_*TTT*_ represents the control objective function; *l*_*i*_ represents one path that belongs to the path set *Y* = (*l*_1_,*l*_2_,….,*l*_*p*′_); $F_{l_{i}}(x_{l_{i}}(k),u_{l_{i}}(k),d_{l_{i}}(k),k)$ represents the travel time of path *l*_*i*_ during period *k*; $x_{l_{i}}(k)$ represents the state variable; $u_{l_{i}}(k)$ represents the control variable; $v_{l_{i}}(k)$ represents the control variable of the lower-level programming model; $d_{l_{i}}(k)$ represents the disturbance variable.

**Fig 3:**
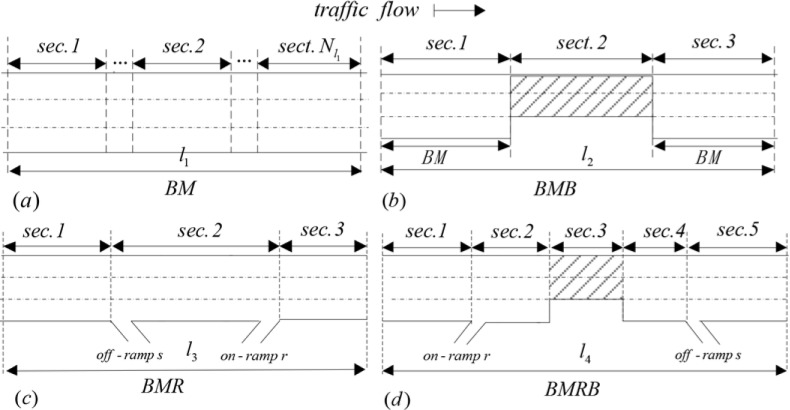
Road alignments: (a) basic mainline section; (b) basic mainline section involving bottleneck; (c) basic mainline section including ramp; (d) basic mainline section including both ramp and bottleneck.

In the upper-level programming model, the accuracy of the travel time calculated affects the traffic control effect. According to the difference of freeway type, the freeway road can be classified into four groups, which are the basic mainline section (BM) *l*_1_, the basic mainline section involving bottleneck (BMB) *l*_2_, the basic mainline section including ramp (BMR) *l*_3_ and the basic mainline section including ramp and bottleneck (BMRB) *l*_4_. A simple example is shown in Fig 3.

If there are no ramps or bottlenecks on the freeway path as presented in Fig 3(A), the traffic flow is mainly influenced by the external environment and internal disturbance. The travel time calculation model can be described by the total time of traffic flows through each path in the freeway network. The travel time of *l*_1_ can be expressed by Eq (11).

$$F_{l_{1}}(k)=\sum_{i=1}^{N_{l_{1}}}\frac{\rho_{l_{1},i}(k)\lambda_{l_{1}}\Delta x_{l_{1}}}{q_{l_{1},i}(k)},$$ (11)

where $F_{l_{1}}(k)$ represents the travel time of *l*_1_; $N_{l_{1}}$ represents the number of elements; $\rho_{l_{1},i}(k)$ represents the traffic density; $\Delta x_{l_{1}}$ represents the length of *l*_1_; $\lambda_{l_{1}}$ represents the number of lanes; $q_{l_{1},i}(k)$ represents the traffic flow.

If there is a bottleneck on the freeway path without ramps, e.g. Fig 3(B), we call basic mainline section involving bottleneck. We select a simple path as an example to describe the travel time of *l*_2_. In Fig 3(B), the path can be divided into three main regions, including two mainline segments and one bottleneck segment. According to the basic method of the travel time calculation in Eq (11) and the change of segment lanes in *l*_2_, we can calculate the travel time of *l*_2_ based on Eq (12).

$$F_{l_{2}}(k)=\sum_{i=1}^{N_{l_{2},1}}\frac{\rho_{l_{2},1,i}(k)\lambda_{l_{2}}\Delta x_{l_{2},1,i}}{q_{l_{2},1,i}(k)}+\sum_{i=1}^{N_{l_{2},3}}\frac{\rho_{l_{2},3,i}(k)\lambda_{l_{2}}\Delta x_{l_{2},3,i}}{q_{l_{2},3,i}(k)}+\sum_{i=1}^{N_{l_{2},2}}\frac{\rho_{l_{2},2,i}(k)\lambda_{l_{2},2}\Delta x_{l_{2},2,i}}{q_{l_{2},2,i}(k)},$$ (12)

where $F_{l_{2}}(k)$ represents the total travel time of vehicles on *l*_2_; $N_{l_{2},1}$, $N_{l_{2},2}$, and $N_{l_{2}3}$ represent the number of elements on segment 1, segment 2, and segment 3, respectively; $\rho_{l_{2},1,i}(k)$ and $q_{l_{2},1,i}(k)$ represent traffic density and traffic flow of the basic element *i* on the segment 1, segment 2, and segment 3, respectively; $\Delta x_{l_{2},i}$ represents the length of element and $\lambda_{l_{2}}$ represents the number of lanes.

Ramp, as an important part of the freeway network, connects the freeway network with other roads. According to the variety of ramps, the BMR can be divided into three groups, including on-ramp, off-ramp, and both on-ramp and off-ramp. We select a path (see Fig 3(C)), involving one on-ramp and one off-ramp, as an example to build the calculation model of travel time for *l*_3_, which is presented in Eq (13).

$$F_{l_{3}}(k)=\sum_{i=1}^{N_{l_{3,1}}}\frac{\rho_{l_{3},1,i}(k)\lambda_{l_{3}}\Delta x_{l_{3},1,i}}{q_{l_{3},1,i}(k)}+\sum_{i=1}^{N_{l_{3,2}}}\frac{\rho_{l_{3},2,i}(k)\lambda_{l_{3}}\Delta x_{l_{3},2,i}}{q_{l_{3},2,i}(k)}+\sum_{i=1}^{N_{l_{3,3}}}\frac{\rho_{l_{3},3,i}(k)\lambda_{l_{3}}\Delta x_{l_{3},3,i}}{q_{l_{3},3,i}(k)},$$ (13)

where $F_{l_{3}}(k)$ represents the total travel time of vehicles on *l*_3_; $N_{l_{3},1}$, $N_{l_{3},2}$, and $N_{l_{3},3}$ represent the element number of segment 1, segment 2, and segment 3, respectively; $\rho_{l_{3},1,i}(k)$, $\rho_{l_{3},2,i}(k)$, and $\rho_{l_{3},3,i}(k)$ represent traffic density and traffic flow of the basic element *i* on the segment 1, segment 2, and segment 3, respectively.

The last case is the BMRB including ramp and bottleneck, and there are some complicated conditions in this region. One case of BMRB, including one on-ramp, one off-ramp, and one bottleneck, is considered in this paper, which can be seen in Fig 3(D). Based on the characteristics of traffic composition and operation, this case can be divided into five segments, involving BM, BMB, and BMR. Therefore, the travel time of *l*_4_ can be calculated with Eq (14).

$$\begin{array}{l} F_{l_{4}}(k)=\sum_{i=1}^{N_{l_{4},1}}\frac{\rho_{l_{4},1,i}(k)\lambda_{l_{4}}\Delta x_{l_{4},1,i}}{q_{l_{4},1,i}(k)}+\sum_{i=1}^{N_{l_{4},4}}\frac{\rho_{l_{4},4,j}(k)\lambda_{l_{4}}\Delta x_{l_{4},4,j}}{q_{l_{4},4,j}(k)}+\sum_{i=1}^{N_{l_{4},2}}\frac{\rho_{l_{4},2,i}(k)\lambda_{l_{4}}\Delta x_{l_{4},2,i}}{q_{l_{4},2,i}(k)}+\\ \;\sum_{i=1}^{N_{l_{4},3}}\frac{\rho_{l_{4},3,i}(k)\lambda_{l_{4},3}\Delta x_{l_{4},3,i}}{q_{l_{4},3,i}(k)}+\sum_{i=1}^{N_{l_{4},5}}\frac{\rho_{l_{4},5,i}(k)\lambda_{l_{4}}\Delta x_{l_{4},5,i}}{q_{l_{4},5,i}(k)} \end{array},$$ (14)

where $F_{l_{4}}(k)$ represents the travel time of *l*_4_; $N_{l_{4},1}$, $N_{l_{4},2}$, $N_{l_{4},3}$, $N_{l_{4},4}$, and $N_{l_{4},5}$ represent the number of element from segment 1 to segment 5, respectively; $\rho_{l_{4},i}(k)$ and $q_{l_{4},i}(k)$ represent traffic density and traffic flow of element *i*, respectively; $\lambda_{l_{4}}$ and $\lambda_{l_{4},3}$ represent the number of lanes of the basic segment and bottleneck segment, respectively.

From the perspective of decision-makers, the first objective of the upper-level programming model represents the travel time of freeway network, and the second objective represents the traffic loading distribution of the network. The managers balance traffic flow in the freeway network based on the traffic operation and demands. Since some special conditions considered in this paper, such as bottleneck region and merging region, it is difficult to use the traditional definition of the traffic load (TLD) to show the actual traffic load. In this paper, the TLD is defined as the ratio between the maximum traffic flow among each section and the minimum capacity of each section in path *l*_*i*_ (see Eq (15)). TLD is a dynamic variable with the traffic conditions of each path changes. The decision about the TLD is complicated and depends on both the traffic conditions and the road type.

$$TLD_{l_{i}}(k)=\frac{q_{l_{i},\max}(k)}{C_{l_{i},\min}},$$ (15)

where $q_{l_{i},\max}(k)$ represents the maximum traffic flow among sections; $C_{l_{i},\min}$ represents the minimum capacity in path *l*_*i*_.

According to Eq (15), we can achieve the traffic load in path *l*_*i*_. The average value of TLD can be shown in Eq (16).

$$T\bar{L}D(k)=\frac{1}{p^{\prime }}\sum_{l_{i}=1}^{l_{p^{\prime }}}TLD_{l_{i}}(k),$$ (16)

where $T\bar{L}D(k)$ represents the average of these paths *l*_1_,*l*_2_,…,*l*_*p*′_.

The second objective function of the upper-level programming model *S*_*TTD*_ is presented in Eq (17) based on Eq (15) and Eq (16).

$$S_{TTD}=\sum_{k\in K}{\left(\sum_{i=1}^{p^{\prime }}{\left(TLD_{l_{i}}(k)-T\bar{L}D(k)\right)}^{2}\right)}^{\frac{1}{2}}.$$ (17)

To equilibrium dimensions of the objective functions, weighting factors are introduced. Therefore, the upper-level optimal control objective function is shown in Eq (18).

$$\begin{array}{l} \min S_{OU}=\alpha_{TTT}\sum_{k\in K}\sum_{i=1}^{p^{\prime }}F_{l_{i}}(x_{l_{i}}(k),u_{l_{i}}(k),v_{l_{i}}(k),d_{l_{i}}(k),k)+\\ \;\alpha_{TTD}\sum_{k\in K}{\left(\sum_{i=1}^{p^{\prime }}{\left(TLD_{l_{i}}(k)-T\bar{L}D(k)\right)}^{2}\right)}^{\frac{1}{2}} \end{array},$$ (18)

where *S*_*OU*_ represents the objective function of the upper-level programming model; *α*_*TTT*_ and *α*_*TTD*_ are the weighting factors of *S*_*TTT*_ and *S*_*TTD*_, respectively.

The upper-level programming model should be restricted by the traffic system constraints to meet the demands of the optimal control. The basic constraint conditions can be shown as follows.

$$\begin{array}{l} s.t.\\ q_{l_{i},1}(k+1)=\left(\rho_{l_{i},1}(k)+\frac{T}{\Delta l_{l_{i},1}\lambda_{l_{i},1}}\left[q(k)\varpi_{l_{i}}(k)-q_{l_{i},1}(k)\right]\right)v_{l_{i},1}(k+1)\lambda_{l_{i},1}\\ q_{l_{i}}(k)=\sum_{\mu \in I_{n}}q_{in,\mu }(k)\varpi_{l_{i}}(k)\\ x_{l_{i}}(k+1)=\varphi_{l_{i}}[x_{l_{i}}(k),u_{l_{i}}(k),v_{l_{i}}(k),d_{l_{i}}(k),k]\\ \sum_{i=1}^{p}\varpi_{l_{i}}(k)=1\\ \varpi_{l_{i}}{}_{,\min}\leq \varpi_{l_{i}}(k)\leq \varpi_{l_{i}}{}_{,\max}\\ 0\leq \varpi_{l_{i}}{}_{,\min}\leq \varpi_{l_{i}}{}_{,\max}\leq 1\\ 0\leq \sum_{i=1}^{p^{\prime }}q_{l_{i},N_{l_{i}}}(k)\leq Q_{out,net,cap}\\ 0\leq q_{l_{i}}(k)\leq Q_{l_{i}}-\Delta Q_{l_{i}} \end{array},$$

where *ξ* represents the compliance rate, 0 ≤ *ξ* ≤ 1; $\varpi_{nor,}{}_{l_{i}}(k)$ represents nominal splitting of *l*_*i*_ without guidance information; $\varpi_{VMS,}{}_{l_{i}}(k)$ represents the splitting rate choosing path *l*_*i*_ under the guidance information; $\sum_{l_{i}=l_{1}}^{l_{p^{\prime }}}\varpi_{l_{i}}(k)=1$.

Considering the driver compliance rates, the splitting rate $\varpi_{l_{i}}(k)$ can be presented by Eq (19).

$$\varpi_{l_{i}}(k)=(1-\xi )\varpi_{nor,}{}_{l_{i}}(k)+\xi \varpi_{VMS,}{}_{l_{i}}(k).$$ (19)

In addition, the splitting rate $\varpi_{l_{i}}(k)$ also can be shown as Eq (20).

$$\varpi_{l_{i}}(k)=q_{l_{i}}(k)/\sum_{\mu \in I_{n}}q_{in,\mu }(k),$$ (20)

where *I*_*n*_ is the connection segment set on the original note upstream; $q_{l_{i}}(k)$ is traffic flow of *l*_*i*_.

According to the Eq (19) and Eq (20), we can get the traffic volume of *l*_*i*_ that can be presented in Eq (21).

$$q_{l_{i}}(k)=\sum_{\mu \in I_{n}}q_{in,\mu }(k)((1-\xi )\varpi_{nor,l_{i}}(k)+\xi \varpi_{VMS,}{}_{l_{i}}(k)).$$ (21)

### Formulation of the lower-level optimal model

The bi-level programming optimal model focuses on the stability and optimization of each path in the test network. Building the upper-level programming model, we consider the problem of the network traffic balance and the traffic flow distribution of the freeway network. In the lower-level programming model, we adopt the VSL control method proposed by Ma et al. [11, 24] to solve the traffic congestion in path *l*_*i*_. Therefore, the primary goal of the lower-level programming model is to ensure the optimization of traffic volume and the travel time in path.

We define the traffic condition of no-control as a control condition. Hence, the control condition parameter $SW_{l_{i}}$ is given as

$$SW_{l_{i}}=\left\{ \begin{array}{l} 0\;non-control,\\ 1\;otherwise; \end{array}\right..$$ (22)

Thus, the objective function of each path in the regional network is showed in this paper as Eq (23).

$$S_{l_{i}}=SW_{l_{i}}\sum_{k\in K}R_{l_{i}}(x_{l_{i}}(k),u_{l_{i}}(k),v_{l_{i}}(k),d_{l_{i}}(k),k).$$ (23)

The basic constraint conditions of the lower-level programming model can be shown as follows.

$$\begin{array}{l} s.t.\\ q_{l_{i}}(k)=\sum_{\mu \in I_{n}}q_{in,\mu }(k)\left(\left(1-\xi )\varpi_{nor,l_{i}}(k)+\xi \varpi_{VMS,}{}_{l_{i}}(k\right)\right)\\ x_{l_{i}}(k+1)=f_{l_{i}}(x_{l_{i}}(k),u_{l_{i}}(k),v_{l_{i}}(k),d_{l_{i}}(k),k)\\ g(x_{l_{i}}(k),u_{l_{i}}(k),v_{l_{i}}(k),d_{l_{i}}(k),k)\leq 0\\ k=1,2,\ldots ,k_{p} \end{array}.$$

## Application results

In this section, we illustrate the efficiency and robustness of the optimization approach proposed in this paper through two ways. First, we adopt the numerical analysis method to test the sensitivity of the optimization approach based on two different types of networks. In addition, a common and typical network is used as the test network for case study.

### Sensitivity analysis

**Fig 4:**
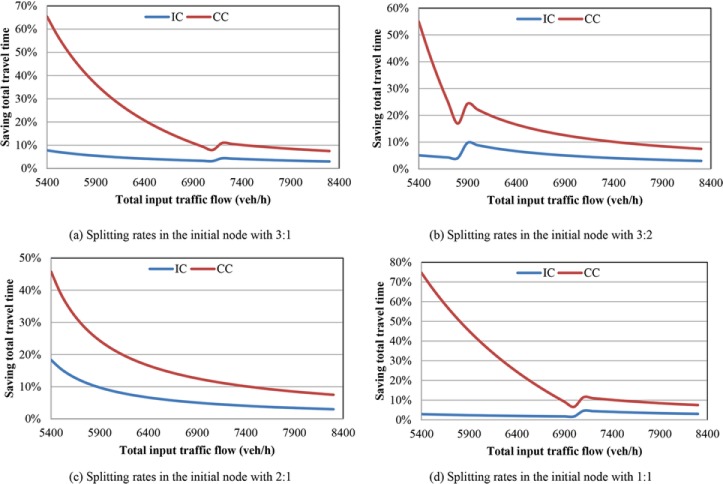
Improvement in terms of saving total travel time with bottlenecks. (a) Splitting rates in the initial node with 3:1. (b) Splitting rates in the initial node with 3:2. (c) Splitting rates in the initial node with 2:1. (d) Splitting rates in the initial node with 1:1.

**Fig 5:**
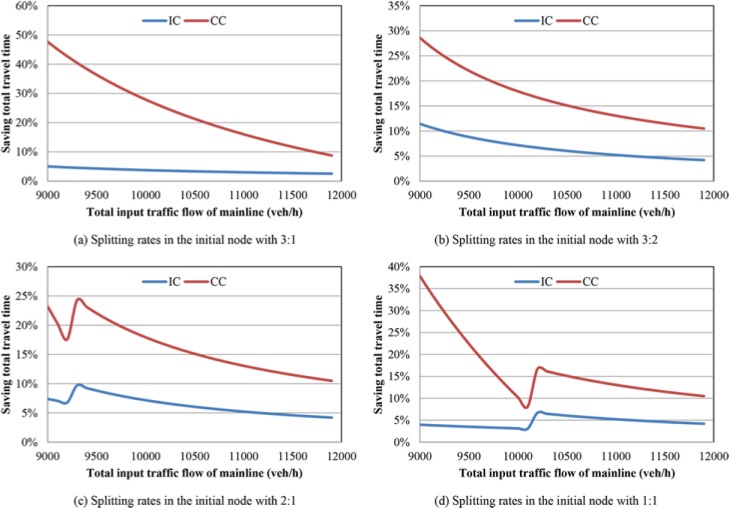
Improvement in terms of saving total travel time with merging regions. (a) Splitting rates in the initial node with 3:1. (b) Splitting rates in the initial node with 3:2. (c) Splitting rates in the initial node with 2:1. (d) Splitting rates in the initial node with 1:1.

We design a series of numerical analysis to study the changes of total travel time obtained from the coordinated control (CC) proposed in this paper and independent control (IC) in previous researches, see [11, 24], in two cases: with bottlenecks or merging regions. It is assumed that there are two paths in the freeway network with the same length 10 km. The path 1 has three lanes while the path 2 has two lanes. The bottlenecks locate at the last 1 km in the two paths respectively. The saturated traffic flow in one lane is 1800 veh/h/lane, and the speed of free traffic flow is 100 km/h. In the numerical analysis, the variable is the input of traffic flow, which is assumed as constant in one experiment. In the independent control, the splitting rates in the initial node are fixed with four settings (input traffic flow to path 1: input traffic flow to path 2) 3:1, 3:2, 2:1 and 1:1. In the proposed coordinated control, the splitting rates in the initial node are obtained by solving the objective function Eq (18). The numerical analysis results with two bottlenecks are shown in Fig 4 and the numerical analysis results with two merging regions are shown in Fig 5.

As shown in the Fig 4, the saving total travel time by coordinated control and independent control compared with no-control case are denoted with red and blue lines respectively. The total input traffic flows of mainline at the initial node are changing from 5400 veh/h to 8400 veh/h. Fig 4(A), 4(B), 4(C), and 4(D) show the saving total travel time under different splitting rates in the initial node. According to the Fig 4, it can be seen that, using the coordinated control, the total travel time can be saved greatly, and along with increase of total input traffic flow, the saving total travel time decreased gradually. Although, using the independent control method can improve the travel speed compared with no-control case, the improvement is relatively small, less than 10%. As shown in the Fig 4(A), 4(B) and 4(D), the lines are not monotone decreasing. When the input traffic flow is relatively large, the two bottlenecks in both paths are activated. The saving total travel time is improved because of the developed the traffic efficiency. When the input traffic flow is relatively small, the improvement of saving total travel time is mainly contributed by proper dynamic splitting rates with coordinated control. Therefore, we can obtain that the coordinated control method performs better than independent control method. When the input traffic flow is small, the coordinated control method can obtain more proper splitting rates in the initial node dynamically and avoid activating the bottlenecks. In addition, when the input traffic flow is relatively large, both the bottlenecks activated, the coordinated control method can improve traffic efficiency greatly.

As shown in the Fig 5, the x-axis refers to the total input traffic flow of mainline (the input traffic flow of on-ramp is constant with 800 veh/h and the off-ramp proportion of mainline traffic flow is 10%), while the y-axis means saving total travel time. The red line and blue line denote the saving total travel time using coordinated control and independent control compared with no-control case respectively. According to the numerical analysis results shown in the figure, the coordinated control can save more travel time than using independent control method. When the total input traffic flow is relative large, the difference of saving total travel time between the two control methods is relatively large with about 20%. With the increase of input traffic flow, the difference changes to be close and stable eventually with about 7%. Similar to the Fig 4, the lines in the Fig 5(C) and 5(D) do not monotone decrease but increase a little in the middle. At the left part the coordinated control can save total travel time greatly because of obtaining proper splitting rates in the initial node and avoiding activating the bottleneck of merging region. When the total input traffic flow is relatively large, the saved total travel time is mainly contributed by increasing the traffic efficiency of merging region.

### Experimentation design and test network

**Fig 6:**
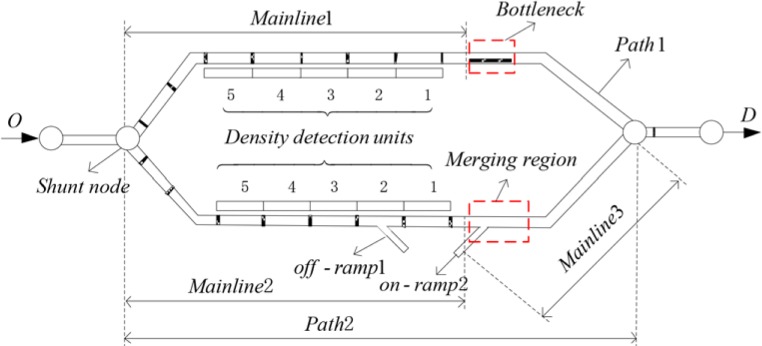
Test freeway network.

To demonstrate the effectiveness of the optimal control approach proposed in this paper, three cases involving the no-control, independent control, and coordinated control methods are applied in the experiment. In particular, the settings of the above scheme are designed as follows: the no-control case is a case without any additional traffic control; independent control case adopts the control approach from the previous researches which are [11, 24]; the coordinated control case adopts the optimal control approach proposed in this paper. To achieve the purpose of this study, we select a simple and ubiquitous freeway regional network as test network, which is presented in Fig 6.

There are two paths in the test freeway network: one represents the bottleneck path BMB, while the other represents the BMR, which are path 1 and path 2, respectively. During the peak period, the traffic congestion phenomenon appears in path 1 and path 2. Moreover, the congestion in path 1 is caused by the bottleneck and the congestion in path 2 mainly appears in the merging region. For ease of analysis and discussion, the upstream mainlines of the bottleneck in path 1 and merging region in path 2 are defined as mainline 1 and mainline 2, respectively.

Furthermore, we have the network parameters: the hypothetic test freeway network has a simple structure and the test time horizon of the test is 5 h. The mainline 1 has three lanes; the bottleneck region and path 2 have two lanes, respectively; ramp 1 and ramp 2 have 1 lane, respectively. The capacity of the test network is 1800 veh/h/lane. The freeway mainline that consists of the upstream mainline of point O and the downstream mainline of point D is composed of four traffic lanes with a legal speed limit of 100 km/h, and the on-ramp own per lane with a legal speed limit of 40 km/h. The model discrete time step length is *T* = 10s. The traffic flowing into the network is shown in Table 1.

**Table 1:** Traffic flow input in test network.

Time	1H	2H	3H	4H	5H
Total flow (veh/h)	5000	7000	6500	7000	5000
Ramp flow (veh/h)	800	800	900	800	800

### Results analysis

**Fig 7:**
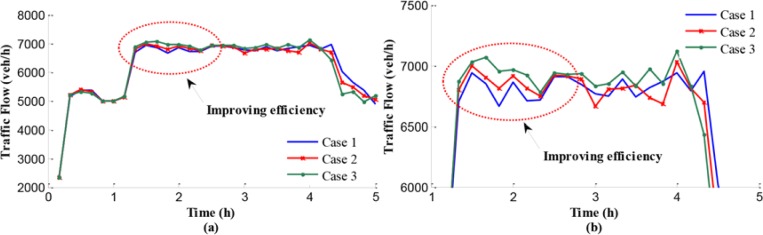
Outflows of the test network: (a) shows the traffic flow during the entire simulation time; (b) represents the detailed information of traffic flow during peak hours.

The studied regional network is formulated on a simulation platform. In this section, we show the simulation results under three different conditions: the no-control case, independent control case, and the coordinated control case. The evaluation results are presented in the following. Freeway network outflow is a major performance index for evaluation of the proposed control approach. Traffic outflows of the test network under the no-control case, independent control case, and the coordinated control case are shown in Fig 7.

As can be seen in Fig 7, the traffic outflow from the test network in the coordinated control case is higher than that under the no-control case and the independent control case. In the first hour, the bottleneck is not activated, and the vehicles can flow into the downstream directly without any delay. During peak hours, the traffic demand is larger than the capacity of freeway network, that the input traffic flow is larger than 7200 veh/h, which can be seen in Fig 7(B). The bottlenecks are activated, where outflow is smaller than the capacity because of chaos of vehicles operation. Although the independent control is applied in the freeway networks in case 2, the outflow is still smaller than it with using coordinated control due to reasonable splitting rates given in case 3. Specifically, at around 1.4 h, a significant drop in the merging region outflow curve can be seen under the no-control case due to the traffic problem activation leading to the decline of total efficiency. In comparison, the traffic outflow from the network in the coordinated control case decreased slightly. Furthermore, compared with no-control case, the total outflows raise about 1900 vehicles in coordinated control case and the peak time reduces 0.34 h, while the independent control only reduces the peak time about 0.15 h.

Travel time is an important index to evaluate the efficiency of the test network and the test results are presented in Table 2. The first column is average travel time of vehicles, and the second and third columns present absolute difference and relative difference of travel time compared with no-control case respectively. As shown in this table, compared with no-control case, we can see that the values of travel time have obvious improvement in independent control case and coordinated control case. In the independent control case, the saving average travel time is about 11 s, while in the coordinated control case performs much better saving average travel time about 44 s. In terms of relative difference, the coordinated control approach proposed in this paper can improve the traffic efficiency with 12.3%, while the improvement is 3.1% using independent control approach. Although both the control methods can improve the traffic efficiency, the coordinated control method can save more travel time compared with independent control method.

**Table 2:** Travel time in three test cases.

**Cases**	**Average travel time (s)**	**Difference compared with no-control case (s)**	**Improvement compared with no-control case (%)**
**No-control case**	357.8	-	-
**Independent control case**	346.6	11.2	3.1
**Coordinated control case**	313.9	43.9	12.3

#### Test results analysis of no-control

**Fig 8:**
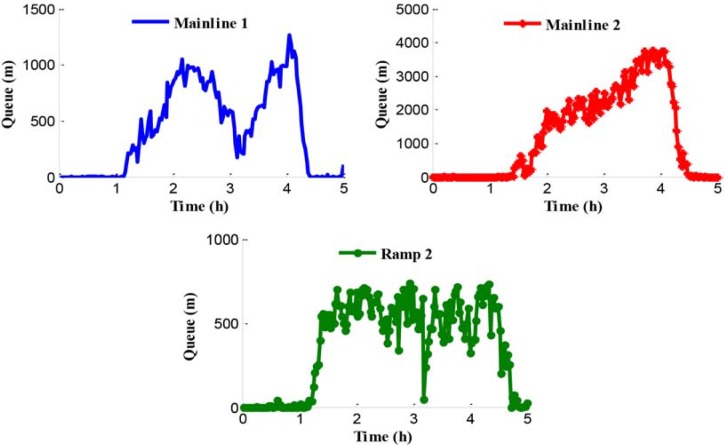
Queue procession in the test network.

The traffic problem can be presented through the analysis of the network traffic operation under the no-control condition. Since the legal speed limit value is adopted in the no-control case, traffic in this simulated system obeys self-organization. The queues in the test network are shown in Fig 8. Experimental results show that the queues in mainline 1 and ramp 2 appear at 1.2 h and the queue in mainline 2 appears at 1.4 h. Furthermore, the reason of the queue in mainline 1 is that the inflow of path 1 is close to the capacity of bottleneck during the peak period and the bottleneck is activated. The traffic congestions in mainline 2 and ramp 2 are caused by the traffic disturbance in the merging region during the peak period.

**Fig 9:**
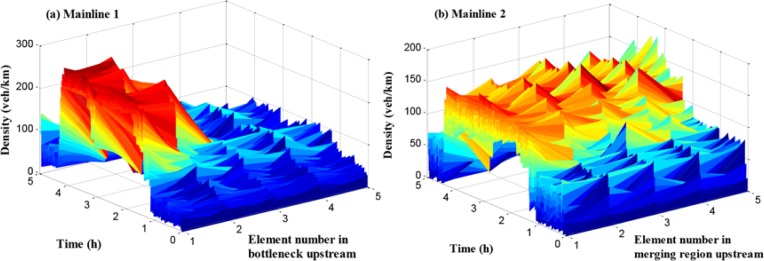
The traffic density of mainline 1 and mainline 2.

Fig 8 shows an obvious difference of the starting time of queue in mainline 2. There is a certain queue time delay under the no-control case compared with ramp 2. The reason for this is that more rights of way are given to the mainline vehicles flowing into the merging region in the no-control case during the peak period. Fig 9 shows the density in mainline 1 and mainline 2 when the no-control approach is applied.

Figs 8 and 9 show the basic parameters extracted from the no-control case, which would provide further support for the analysis of traffic operation in the test network. At 1.7 h, the congestion in the off-ramp 1 and the existing traffic disturbance in the merging region result in a visible decreasing value of the outflow in the mainline 2.

**Fig 10:**
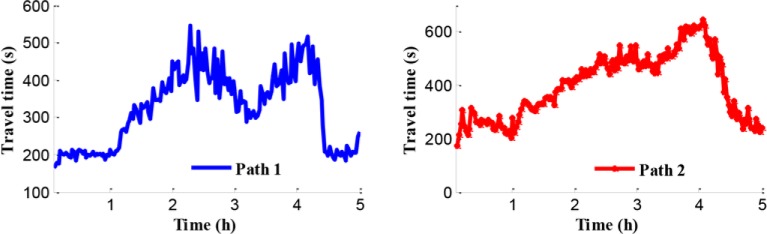
Travel time.

Fig 10 shows the average travel time of path 1 and path 2 during the entire simulation time. By analyzing this information among Figs 8, 9 and 10, it becomes clear that during peak period, heavy traffic flows in both the merging region and the bottleneck region result in aggravated congestion on the freeway network. Consequently, during 1.4 h to 4.3 h, the average travel times that drivers spent in path 1 and path 2 are 333 s and 473 s, respectively.

#### Test results analysis of independent control

**Fig 11:**
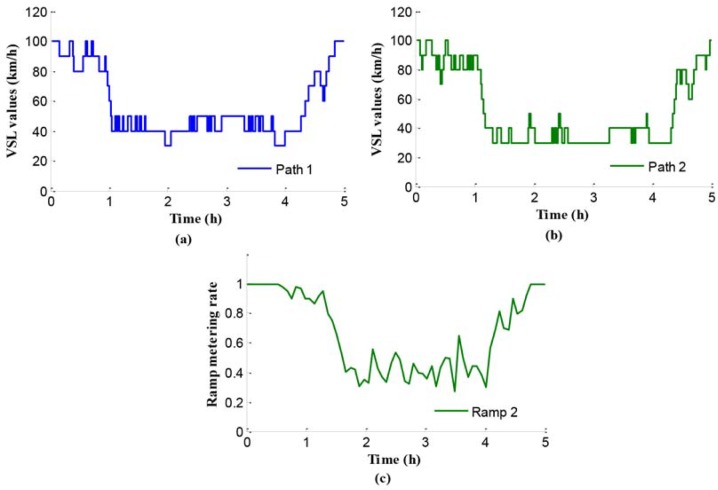
Control parameters in case 2.

Fig 11(A) presents the VSL values of path 1 using independent control method. The x-axis refers to time and the y-axis means the limits values. At the beginning, the average traffic flow is lower than freeway networks capacity, so that the values of speed limits are relatively large without obvious influence to the mainline traffic flow. Later, along with the increase of input traffic flow, a set of low speed limits values are selected in order to maintain the efficiency of traffic flow at the bottleneck with high level.

In the path 2, there is a merging region controlled by the VSL control integrated with ramp metering. Fig 11(B) and 11(C) present the values of variable speed limits and ramp metering rate respectively. From Fig 11(B) and 11(C), we can see that at the beginning both the speed limits control and ramp metering are not triggered because of low input traffic flow. Along with the increase of input traffic flow, a set of low VSL values and ramp metering rates are selected, in order to maintain the traffic efficiency at the merging region. Especially, the ramp metering rates are quite small for providing more right of way to the vehicles on mainline, because of more vehicles on the mainline.

Using the independent control approach, the traffic flow is controlled by VSL control and ramp metering, and the control effects can be reflected by queue length, travel time, and traffic density.

**Fig 12:**
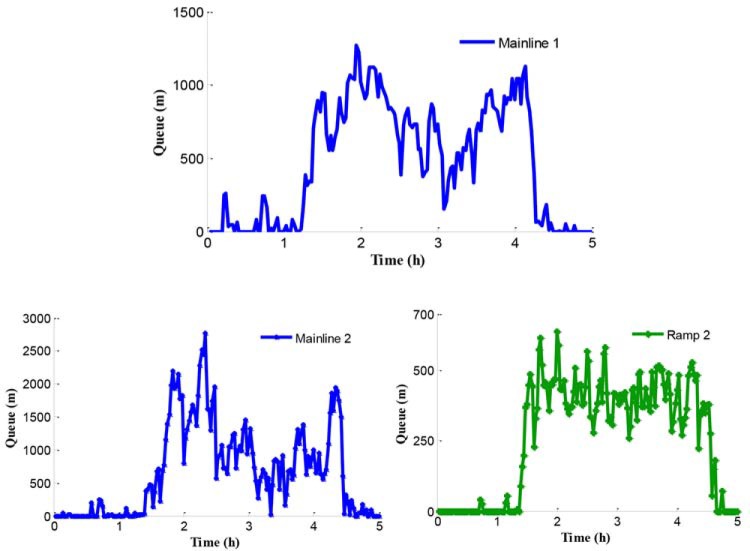
Queue in the test work.

First we can see the queue in the freeway network under the independent control from Fig 12. All the queues on mainline 1, mainline 2, and on-ramp begin to increase at about 1.5 h. During peak period, the queue length on mainline 1 fluctuates around 1000 m, while the queue length on mainline 2 reaches between 1500 m to 2000 m, and the queue length at the on-ramp is a little smaller than 500 m.

**Fig 13:**
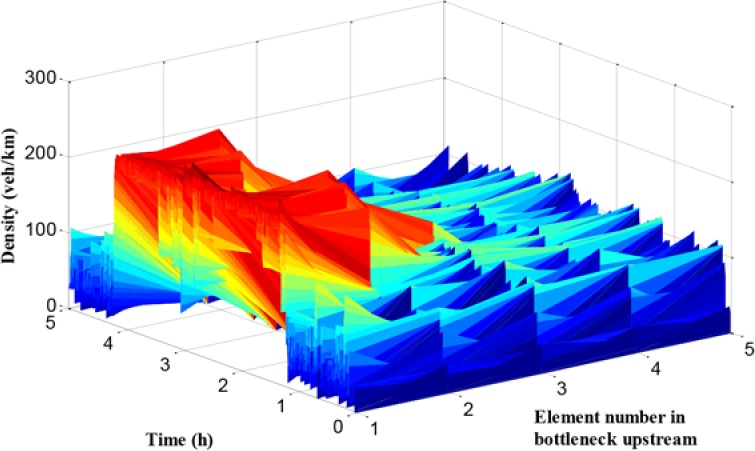
Density in each unit of mainline 1.

As presented in Fig 13, the traffic flow density of path 1 in each cell is given. The x-axis refers to the number of cells, and the y-axis means the simulation time, while the z-axis denotes traffic density. It can be seen that the traffic density during off-peak hours is relatively small with about 50 veh/km/lane, while it reaches more than 200 veh/km/lane during peak period, and the density at cell 1 and cell 2 is much larger than it in backward cells. However, compared with the no-control case, the density distribution is more balanced in the cells and the maximum density is lower.

**Fig 14:**
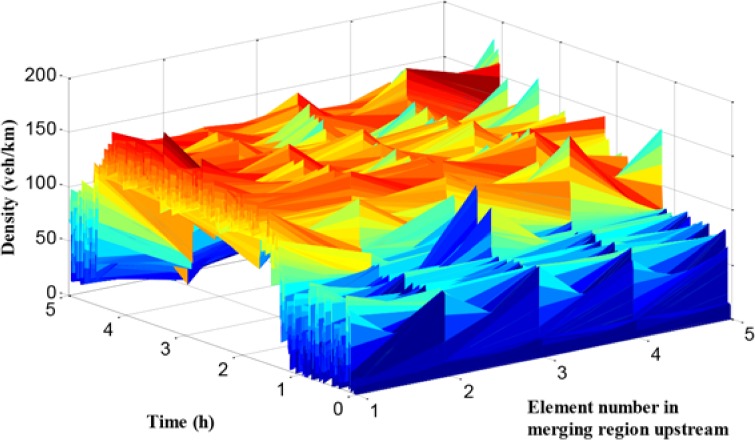
Density in each unit of mainline 2.

In Fig 14, the traffic flow density of path 2 in each cell is given. Compared with the traffic density figure in path 1, the density values of path 2 during peak period in all cells are large, which means that the traffic demand of path 2 is higher than path 1. The traffic condition in path 2 is worse than it in path 1. These results are consistent with the conclusion of travel time comparison.

**Fig 15:**
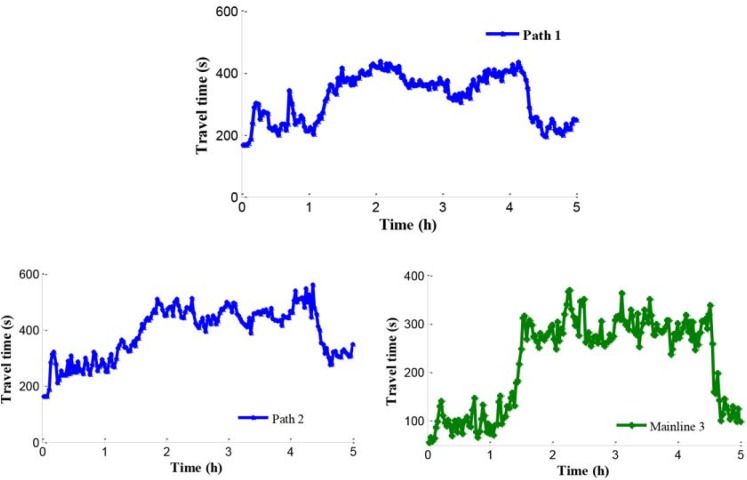
Travel time of path 1, path 2 and mainline 3.

Fig 15 shows average travel time of vehicles on path 1, path 2, and mainline 3 (from on-ramp to the destination). Although the lengths of path 1 and path 2 are almost the same, the travel time of path 2 is larger than it of path 1. It means that the travel speed on path 1 is larger than it on path 2. The travel time of on-ramp increases greatly along with the increase of input traffic flow because the ramp metering rate is relatively low.

#### Test results analysis of coordinated control

**Fig 16:**
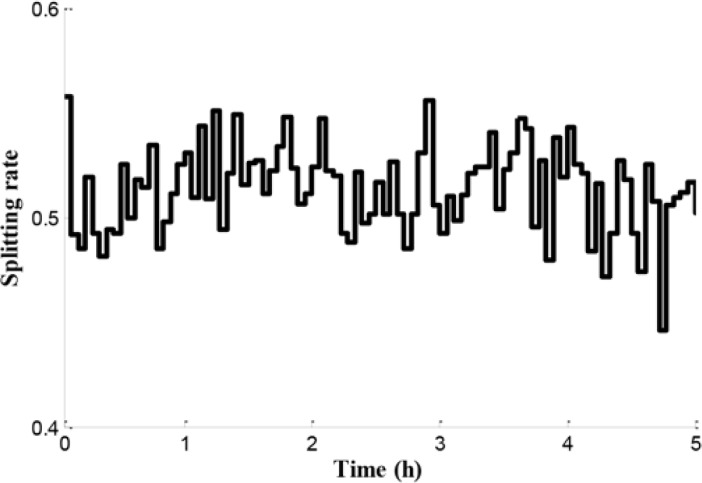
Splitting rates in the initial node O.

The coordinated control approach proposed handles the congestion in the freeway network. Particularly, the control strategy can be represented as the cooperation between path control and network control. The splitting rate as a significant parameter can be achieved by the upper-level optimal control model. As shown in Fig 16, the splitting rate is fluctuating and ranges between 0.4 and 0.6.

**Fig 17:**
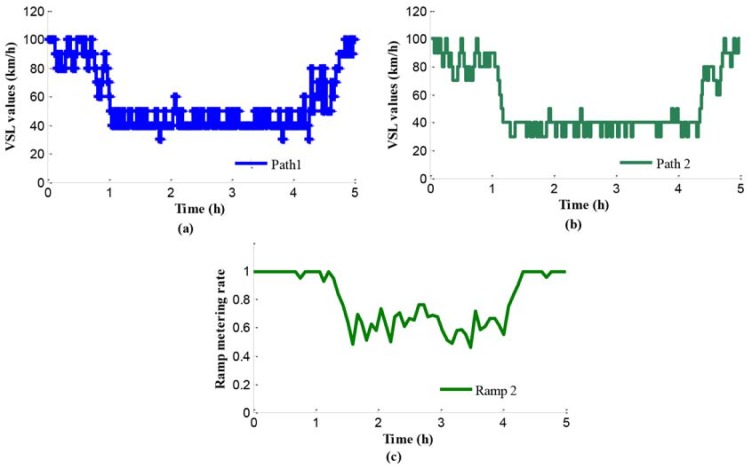
Control parameters in case 3.

In the lower-level programming model, the decision variables include the VSL values in path 1 and the VSL values and ramp metering rates in path 2, all of which are shown in Fig 17. Furthermore, Fig 17(A) and 17(B) show the VSL values, with 10 step-lengths as scale variations. Fig 17(C) shows the RM rates in path 2. During peak period, the VSL values of path 2 are inferior to those of path 1, because ramp metering is adopted in path 2. Especially, when the traffic condition index is higher than the setting threshold, the mainline controllers may select a series of lower VSL values to strain the outflows of mainline 2.

**Fig 18:**
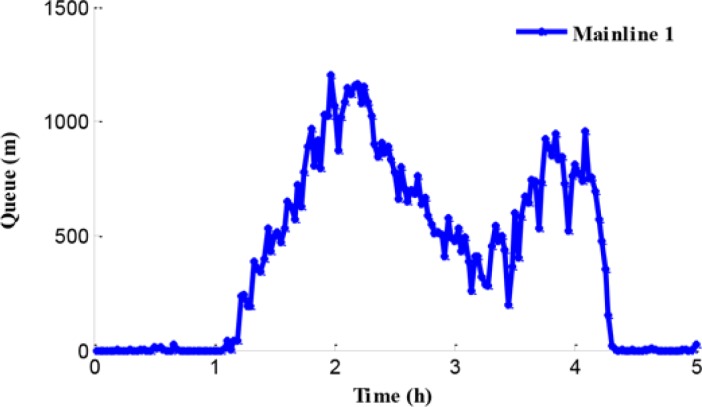
Queue in mainline 1.

**Fig 19:**
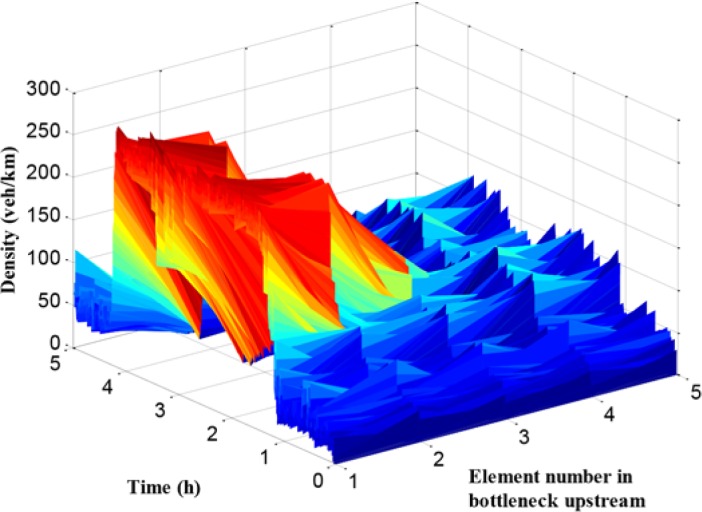
Density in each unit of mainline 1.

When the traffic flow in the upstream region of the bottleneck is higher than the bottleneck capacity, the bottleneck is promptly activated leading to a series of corresponding traffic problems in mainline 1. The queue and the density in mainline 1 are presented in Figs 18 and 19, respectively.

In Figs 18 and 19, the queue and density of mainline 1 always maintain high values during peak period, indicating the existence of severe traffic congestion in mainline 1. The average queue and maximum queue are 639 m and 1020 m, respectively. According to Figs 8, 12 and 18, the queue in mainline 1 has a small extent improvement compared with the no-control case and independent control case in that a series of lower VSL values were adopted in the coordinated control case to relieve traffic congestion. Furthermore, in Figs 9 and 19, the mainline 1 density in the coordinated control case is lower than that of the no-control case. The vehicles in the coordinated control case are in a dynamic queue with a relatively high speed, and the queue time shortens about 0.16 h compared with the no-control case. In addition, in Figs 13 and 19, the density in mainline 1 under coordinated control case is higher than it under independent control case. The result is caused by unreasonable split rate in the independent control case. Although the density in mainline 1 under the coordinated control case is a little larger than that under independent control case, a large improvement is achieved in mainline 2.

**Fig 20:**
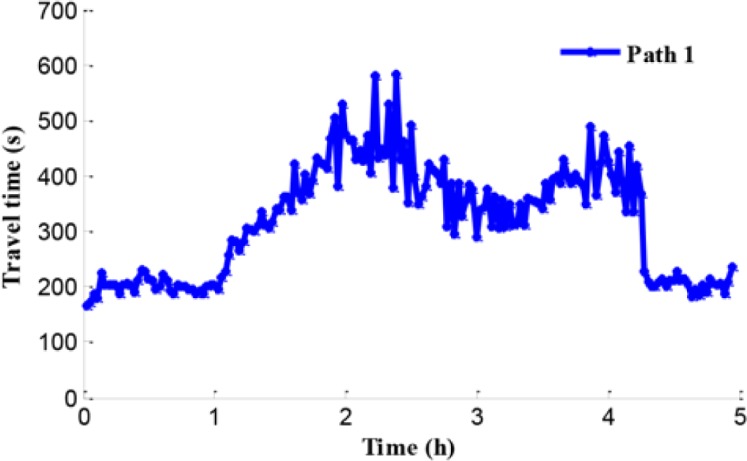
Travel time in the coordinated control case of path 1.

**Fig 21:**
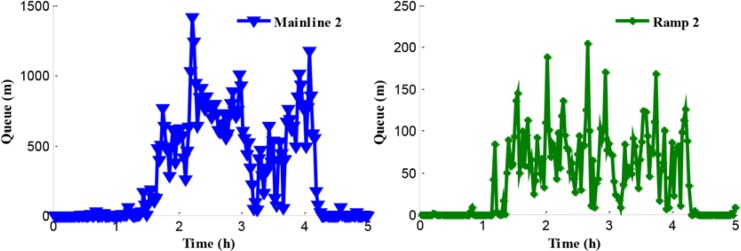
Queue in mainline 2 and ramp 2.

**Fig 22:**
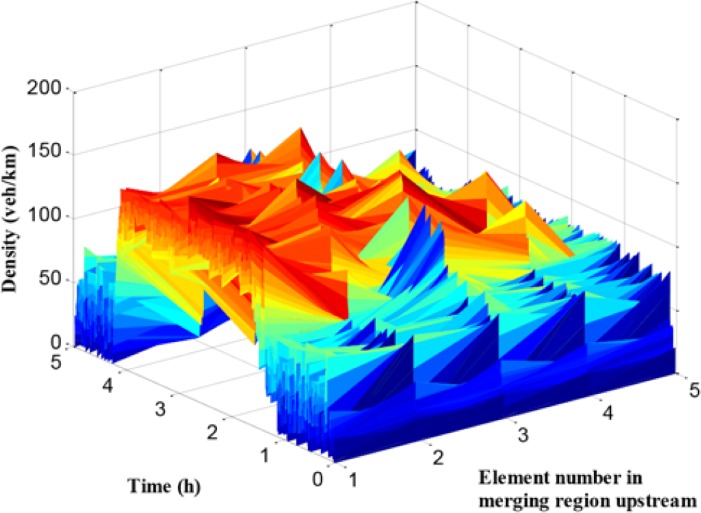
Density in each unit of mainline 2.

Fig 20 shows the travel time in the coordinated control case. According to Figs 10 and 20, the travel time in path 1 has a little improvement of 1.5% in coordinated control case compared with the no-control case. The reason is that the control goal of the coordinated control is enhancing the efficiency of the traffic network rather than improving a single path only. When traffic inflow continues to maintain a high level, the congestion caused by the traffic flow disturbance in merging region occurs in path 2, which is presented in Figs 21 and 22.

According to Figs 8, 9, 21 and 22, the following conclusions can be obtained in comparison with the no-control case.

1. The queue phenomenon in mainline 2 is alleviated and the queue time is reduced. The coordinated control case shortened the average queue length by 75% and the queue time by 0.27 h because of the improvement of the merging region traffic order.
2. The upstream density of mainline 2 is reduced, which means that the dynamic queue vehicles decreased in the coordinated control case. Furthermore, the range of queue in ramp 2 is 0–204 m and the average queue length is 69.5 m. A notable improvement of 86.2% of the queue in ramp 2 can be obtained, while reducing the queue time by 0.42 h.

**Fig 23:**
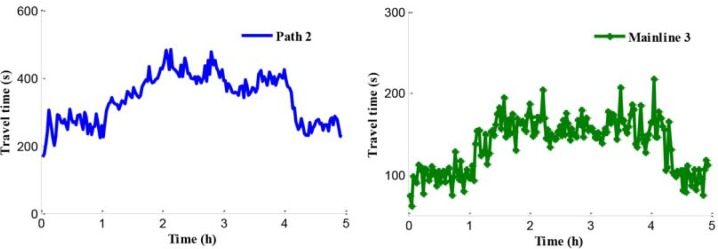
Travel time of path 2 and mainline 3.

In addition, compared with the independent control case, according to Figs 12, 18 and 21, there is a remarkably improvement on the queue in the test network under the coordinated control. Specifically, the extent of improvement about the queue, in mainline 1, mainline 2, and ramp 2, can be expressed by 12%, 53%, and 83% respectively. Seen the queue figures, the queue times in each road are reduced observably. As shown in Figs 10 and 23, there is a large difference between the no-control case and the coordinated control case. Compared with the no-control case, the travel time in path 2 has a better performance than the traffic flow stability enhancement, and mainline 3 shows a strong improvement on travel time of 53%. According to Figs 15 and 23, the travel time in path 3 under coordinated control case is improved about 44%, compared with the independent control case.

## Conclusions and future research

The majority of cities in the world still use route guidance and VSL control. To enhance the traffic efficiency and stability of the traffic network, we propose a novel coordinated control approach that adopts the bi-level optimal model combining VSL, ramp metering, and road guidance. The control approach mainly concerns the optimization and balancing between road network service level and traffic conditions of the traffic network. Specially, the bi-level optimal model proposed in this paper involves two parts: in the upper-level optimal model, we employ the route guidance as basic method concerning the travel time minimum and traffic equilibrium in the network level; and in the lower-level optimal model we pay more attention on the optimization of traffic volume and the travel time in each path of the traffic network.

Furthermore, a real and universal traffic network is selected to verify the proposed approach. The performance of the method is studied using the related traffic parameters, such as travel speed, travel time, and traffic volume. The results demonstrate that the bi-level optimal model proposed in this paper is capable to provide an efficient and flexible transportation environment. More specially, the control strategy that connects the traffic network with each path in the test network is successful in shortening the travel time and improving the traffic conditions. Specifically, in this paper, we consider three different types of traffic networks to evaluate the effect of the optimization control approach proposed in this paper. In detail, two different types of networks, including bottleneck region and merging region separately, are selected to test the sensitivity of the optimization approach. We get some good results using numerical analysis to compare the saving total travel time. Another type of traffic network includes both the bottleneck region and merging region which are set on different paths. Additionally, three cases involving the no-control, independent control, and coordinated control methods are applied in this integrated network, and the effectiveness of the control method is further proved through the simulation. Note that the application type of networks of the optimization control approach including the former test networks, but is not limited to these three types. The scope of application of the optimization method includes different combinations of path types involving common mainline path and the tested paths in this paper. However, some special situations may not been taken into account in this paper, such as the bad weather and the special road alignment.

Although the efficiency and the flexibility of the coordinated control approach are proved in this paper, more complex road networks have not been taken into consideration. In future work, we will apply this control approach proposed in this paper to the variety situation to illustrate the robustness. We also will consider other methods integrated in the control framework to enhance the efficiency.
